# Comparative Analysis of the ASA-PS Score and Clinical Frailty Scale in Predicting Postoperative Intensive Care Unit Requirement in Geriatric Hip Fracture Surgery: A Retrospective Evaluation

**DOI:** 10.3390/jcm15166263

**Published:** 2026-08-13

**Authors:** Dilek Kalaycı, Tuğba Aşkın

**Affiliations:** Department of Anesthesiology and Reanimation, Dr. Abdurrahman Yurtarslan Oncology Training and Research Hospital, University of Health Sciences, Ankara 06200, Turkey; dr.tugba.askin@gmail.com

**Keywords:** Geriatric hip fracture, Clinical Frailty Scale (CFS), American Society of Anesthesiologists Physical Status (ASA-PS), Intensive care unit (ICU) admission, In-hospital mortality, Risk stratification

## Abstract

**Background/Objectives**: Identifying geriatric hip fracture patients who will require postoperative intensive care unit (ICU) admission remains a clinical challenge. The American Society of Anesthesiologists Physical Status (ASA-PS) classification and the Clinical Frailty Scale (CFS) are both used in preoperative risk stratification, yet their comparative utility for this purpose has not been well characterized. **Methods**: This single-center, retrospectively designed study included 243 patients aged 65 years or older who underwent hip fracture surgery between January 2023 and December 2025. The primary outcome was determined as postoperative ICU admission, while the secondary outcomes were in-hospital mortality and postoperative complications. Discriminative performance was assessed by ROC analysis with DeLong pairwise comparison. Multivariable logistic regression analysis was performed to identify independent predictors of ICU admission. **Results**: Postoperative ICU admission occurred in 72.8% of patients. On multivariable analysis, neither ASA-PS nor CFS independently predicted ICU admission. Age (OR 1.052, 95% CI 1.009–1.096; *p* = 0.017), coronary artery disease (OR 3.992, 95% CI 1.581–10.083; *p* = 0.003), and spinal anesthesia (OR 0.363, 95% CI 0.161–0.823; *p* = 0.015) were found to be independent determinants. The addition of either scoring system to this clinical model did not improve discriminative performance (AUC 0.712 vs. 0.709 for both). For in-hospital mortality, CFS demonstrated a markedly superior discriminative ability compared to ASA-PS (AUC 0.801 vs. 0.623; DeLong *p* = 0.047). **Conclusions**: In this study, ASA-PS and CFS demonstrated comparable performance in predicting ICU admission; however, neither scale retained independent predictive value after adjustment for age, coronary artery disease, and type of anesthesia. Moreover, incorporating either score into the existing clinical risk model did not meaningfully improve discriminative performance (AUC: 0.709 vs. 0.712). Conversely, CFS exhibited superior discriminative ability compared to ASA-PS for in-hospital mortality. Given the low number of mortality events (*n* = 13), this finding should be interpreted cautiously as exploratory and warrants confirmation in larger, prospective, multicenter studies.

## 1. Introduction

Geriatric hip fractures have become one of the most common health problems in both anesthesia and surgical practice worldwide, along with the rapidly increasing elderly population. According to United Nations data, the global population aged 65 and older is expected to account for one-sixth of the global population by 2050 [1]. This patient group has an increased risk of postoperative morbidity, mortality, and postoperative intensive care unit (ICU) admission due to decreased physiological reserves, accompanying comorbidities, and high frailty levels. This situation also brings about a significant increase in healthcare system costs [2]. Therefore, predicting which patients will require ICU support in the postoperative period during the preoperative period and careful perioperative risk classifications are critically important to improve outcomes [3].

The American Society of Anesthesiologists physical status classification (ASA-PS) is used in daily practice to assess anesthesia risk. However, this classification focuses on the patient’s comorbidities and does not reflect the patient’s current functional capacity or physiological reserve [4]. Although the ASA-PS was originally developed to classify perioperative physical status rather than to predict specific postoperative outcomes such as ICU admission [4], its inter-rater reliability and predictive performance have been shown to vary considerably across surgical populations [5,6]. Accordingly, its predictive validity for such outcomes in geriatric patients remains underexplored. Recent studies show that ASA-PS classification may be inadequate in predicting postoperative outcomes in geriatric surgical patients [7]. Therefore, especially in geriatric patients, evaluating not only comorbidities but also the patient’s overall frailty status can improve the patient’s postoperative outcomes.

Unlike standard comorbidity indices, preoperative frailty assessment evaluates the patient’s physical, cognitive, and functional reserves, thereby providing a more comprehensive physiological evaluation [8]. Clinical studies demonstrate strong associations between preoperative frailty, mortality and complications [9], and prolonged hospital stay [10]. The Clinical Frailty Scale (CFS) is one of these assessment tools and is an easy and quick method to apply. Kennedy and colleagues demonstrated in their study that CFS is a valid and easy assessment tool for predicting survival in patients with hip fractures [11]. Saetang and colleagues prospectively demonstrated that CFS, together with the mFI-11 and FRAIL scale, is a valid tool for predicting postoperative complications in older non-cardiac surgical patients [12]. However, evidence on the comparative effectiveness of these scores in determining postoperative intensive care need in patients undergoing geriatric hip surgery is still limited.

The purpose of this study was to compare the discriminative performances of ASA-PS and CFS in predicting postoperative ICU admission in a geriatric hip fracture cohort, and to evaluate the incremental predictive value they provide beyond known clinical risk factors. We anticipate that the data obtained from our research will contribute to anesthesiologists’ selection of more sensitive tools in preoperative risk assessment and to the management of these patients by identifying high-risk patients in the early period.

## 2. Materials and Methods

**Figure 1:**
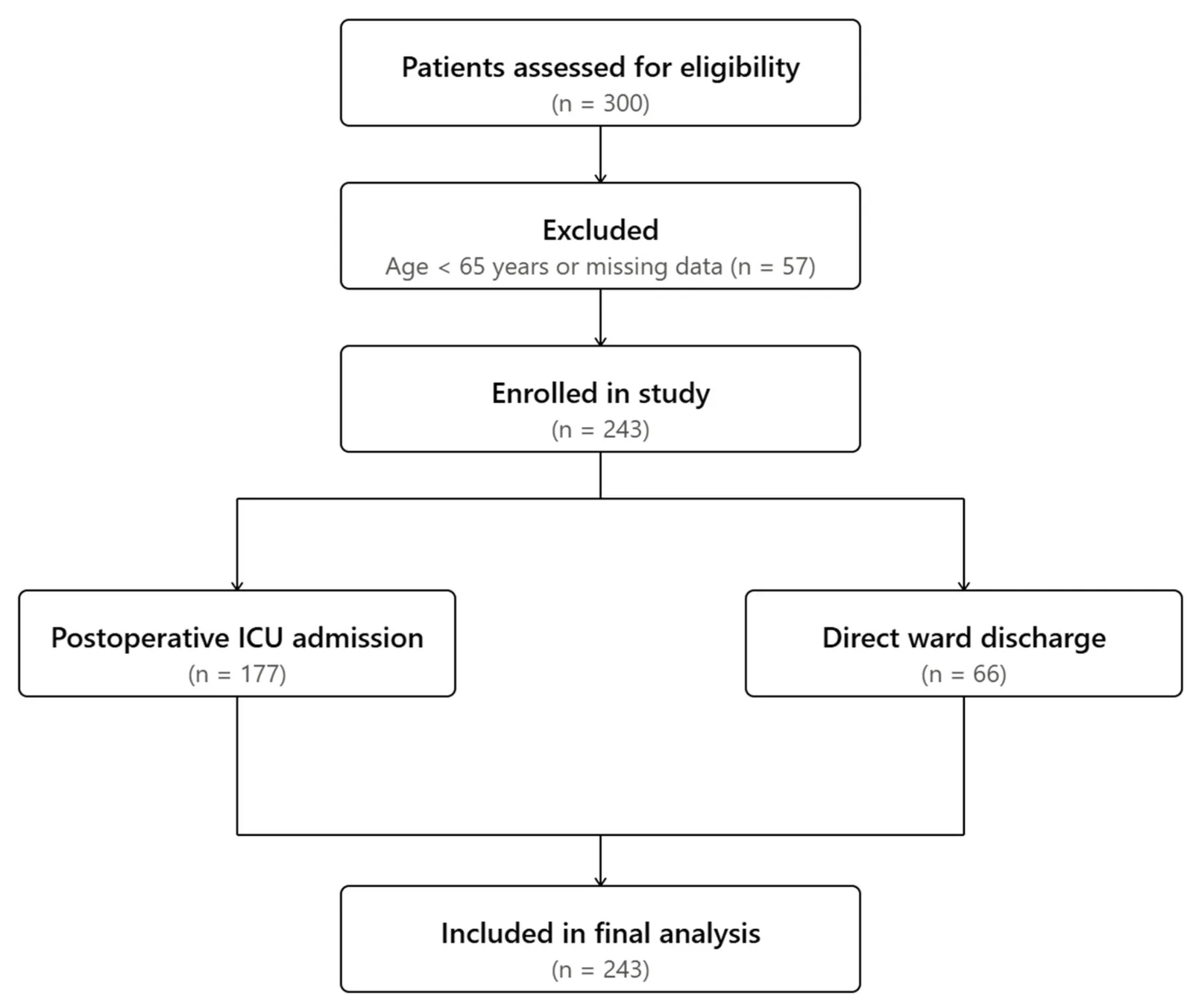
Flow diagram of patient selection.

This single-center, retrospective, observational study included patients aged 65 years or older who underwent urgent or emergency hip fracture surgery at a tertiary care hospital in Ankara, Türkiye, between 1 January 2023 and 31 December 2025. Patients were identified through the hospital information system records. A total of 300 patients who underwent hip fracture surgery were initially screened. Of these, 57 patients were excluded because they were under 65 years of age or had incomplete clinical/laboratory data, and the remaining 243 patients were included in the study (Figure 1).

The urgent group comprised patients placed on the scheduled operative list within the first 24–48 h following the optimization of comorbidities, whereas the emergency group represented cases taken for immediate surgery within the first 6 h due to reasons such as open fractures or neurovascular compromise. Patients with pathological fractures, multiple trauma, acetabular fractures, a history of previous hip fracture surgery, or incomplete data were excluded. Patients were divided into two groups based on whether they were admitted to the postoperative ICU or not. Data were obtained from the hospital information system, anesthesia records, operating room forms, and postoperative follow-up notes. Patients’ demographic characteristics (age, sex), preoperative comorbidities (hypertension, coronary artery disease, congestive heart failure, diabetes mellitus, chronic obstructive pulmonary disease, chronic kidney disease, dementia), preoperative laboratory values (Hemoglobin, creatinine, sodium, albumin, glomerular filtration rate (GFR), platelet, lymphocyte, white blood cell (WBC)), fracture type, anesthesia technique (spinal/general), surgical duration (minutes), intensive care unit admission and length of stay, length of hospital stay, postoperative complications, and mortality were recorded. The ASA-PS classification was obtained directly from the preoperative anesthesia forms. CFS scores were assigned retrospectively by a single experienced anesthesiologist who was blinded to all postoperative outcomes, based on structured review of preoperative anesthesia and nursing documentation. Scoring reflected the patient’s baseline cognitive and physical functions during the two-week period preceding the hip fracture [13]. Inter-rater reliability of retrospective CFS ascertainment was evaluated in a subset of 30 patients selected from the beginning of the study period, consistent with recommended sample sizes for reliability studies [14]. Both raters were blinded to postoperative outcomes at the time of CFS scoring. Agreement was assessed using a two-way random-effects ICC and linearly weighted Cohen’s kappa; the resulting ICC of 0.982 (95% CI: 0.961–0.992, *p* < 0.001) and weighted kappa of 0.957 indicated excellent inter-rater agreement. Patients with a CFS score of 5 or higher were classified as frail, and ASA-PS scores were dichotomized at a threshold of 3 to distinguish patients at higher anesthetic risk. To further characterize the relationship between these two tools, patients were classified into four concordance/discordance groups in an exploratory analysis: Group 1 (ASA-PS 2 with non-frail CFS, *n* = 12; 4.9%), Group 2 (ASA-PS 2 with frail CFS, *n* = 32; 13.2%), Group 3 (ASA-PS 3–4 with frail CFS, *n* = 171; 70.4%), and Group 4 (ASA-PS 3–4 with non-frail CFS, *n* = 28; 11.5%). Postoperative ICU admission was determined by the attending anesthesiologist based on clinical judgment and intraoperative course—including hemodynamic instability, blood loss, surgical duration, and cardiorespiratory complications—without a standardized triage protocol. Due to the study’s retrospective design, these intraoperative factors were not systematically recorded as quantifiable criteria. Postoperative complications were verified through the hospital information system using ICD-10 codes and consultation records requested from the relevant departments. Acute Kidney Injury (AKI) was defined according to the 2012 KDIGO criteria. Postoperative delirium was identified retrospectively through ICD-10 discharge codes and documented psychiatry or neurology consultation records in the hospital information system, as no prospective bedside assessment instrument was applied. Myocardial injury was identified based on cardiology consultations requested upon clinical suspicion, without routine postoperative troponin screening. In-hospital mortality was defined as all-cause death before discharge during the initial admission; post-discharge survival was not followed.

Statistical analyses were performed using IBM SPSS Statistics (Version 24.0; IBM Corp., Armonk, NY, USA). The normality of continuous variables was assessed using the Shapiro–Wilk test. Categorical variables were expressed as counts and percentages, and continuous variables were presented as medians with interquartile ranges (IQR) or means ± standard deviations, according to data distribution. The incremental contribution of each scoring system to the base model was evaluated using the omnibus likelihood ratio test via block-entry binary logistic regression. Receiver Operating Characteristic (ROC) analysis and pairwise Area Under the Curve (AUC) comparisons using the DeLong method were performed with the pROC package in Jamovi (Version 2.6; The Jamovi Project, Sydney, Australia).

The chi-square test or Fisher’s exact test was used to compare categorical variables, and the Mann–Whitney U test or independent samples t-test was applied for continuous variables. The correlation between the ASA-PS score and CFS was evaluated using Spearman’s rank correlation coefficient. The discriminative power of the scores was evaluated using ROC curve analysis, and AUC values were compared using the DeLong method. Optimal cut-off values were determined via the Youden index. To prevent the premature exclusion of clinically relevant predictors, variables demonstrating *p* < 0.10 in univariable screening were entered into a multivariable binary logistic regression model using the Enter method [15]. The variables entered into the multivariable model were: age (*p* = 0.001), coronary artery disease (*p* < 0.001), anesthesia type (*p* = 0.044), CFS ≥ 5 (*p* = 0.034), hemoglobin (*p* = 0.011), and albumin (*p* = 0.004). The events-per-variable ratio for the primary model was approximately 11 (66 ward-managed patients/6 variables), exceeding the accepted threshold of 10 and indicating adequate model stability. Model fit and multicollinearity were checked using standard regression diagnostics. *p*-values < 0.05 were considered statistically significant.

The study protocol was approved by the Clinical Research Ethics Committee of Dr. Abdurrahman Yurtarslan Ankara Oncology Training and Research Hospital (Decision No: 2026-03/69) and was registered at ClinicalTrials.gov (Registry No: NCT07522541). The study was conducted in accordance with the principles of the Helsinki Declaration.

During the preparation of this manuscript, the authors used generative artificial intelligence (GenAI) to assist in the formatting, structuring, and compilation of the data tables. The GenAI tool was utilized solely to organize the presentation of statistical results into tables and to ensure consistent formatting. The authors review and accept full responsibility for the accuracy and integrity of all data presented in the paper.

## 3. Results

A total of 243 patients operated on for geriatric hip fracture were included in the study. The median age of the patients was 81 years (IQR 75–86), and 65.0% (158 patients) were female. Preoperative chronic disease burden was high; hypertension was the most common comorbidity (54.7%, 133 patients), followed by diabetes mellitus (33.3%, 81 patients), chronic kidney disease (25.5%, 62 patients), and coronary artery disease (24.7%, 60 patients). Regarding the anesthesia risk score (ASA-PS), a large proportion of the patients (81.9%, 199 patients) were in the ASA-PS III–IV category, with ASA-PS III constituting the most dense group (48.1%, 117 patients). When the CFS was evaluated, 58.0% of the patients (141 patients) were found to be frail (CFS ≥ 5). Spinal/regional anesthesia was performed in 75.3% of the surgeries (183 patients), and 75.7% (184 patients) were taken to operation under urgent conditions. The preoperative median hemoglobin value of the entire group was 11.1 g/dL (IQR 9.85–12.6), and the albumin value was found to be 35.2 g/L (IQR 31.5–38.1). All these baseline demographic, clinical, and laboratory data of the patients are summarized in Table 1.

Postoperatively, 72.8% of patients were admitted to the ICU, while 27.2% were managed in the ward. Patients admitted to the ICU were significantly older (median 82.0 vs. 76.5 years; *p* = 0.001) and had significantly lower preoperative albumin levels (35.0 vs. 37.1 g/L; *p* = 0.003) and hemoglobin levels (10.8 vs. 11.8 g/dL; *p* = 0.004). Regarding clinical scoring systems, the median CFS score was significantly higher in the ICU group compared to the ward group (6 vs. 5, *p* = 0.021). When evaluated as a categorical threshold, preoperative frailty (CFS ≥ 5) was significantly more prevalent among patients admitted to the ICU than those managed in the ward (62.1% vs. 47.0%, *p* = 0.041). Conversely, no statistically significant difference was observed between the groups regarding the ASA-PS distribution (*p* = 0.224). The presence of coronary artery disease was strongly associated with ICU admission (30.5% vs. 9.1%, *p* < 0.001). Additionally, ICU admission rates were significantly higher among patients who received general anesthesia compared with those who received neuraxial anesthesia (83.3% vs. 69.4%; *p* = 0.044, Fisher’s exact test). Although the ICU and ward groups showed differences in the proportions of high-risk patients (ASA-PS III–IV: 83.6% vs. 77.3%), in-hospital mortality rates (6.8% vs. 1.5%), and overall complication rates (32.2% vs. 21.2%), these differences did not reach statistical significance. Univariable comparisons of all variables are detailed in Table 2.

**Figure 2:**
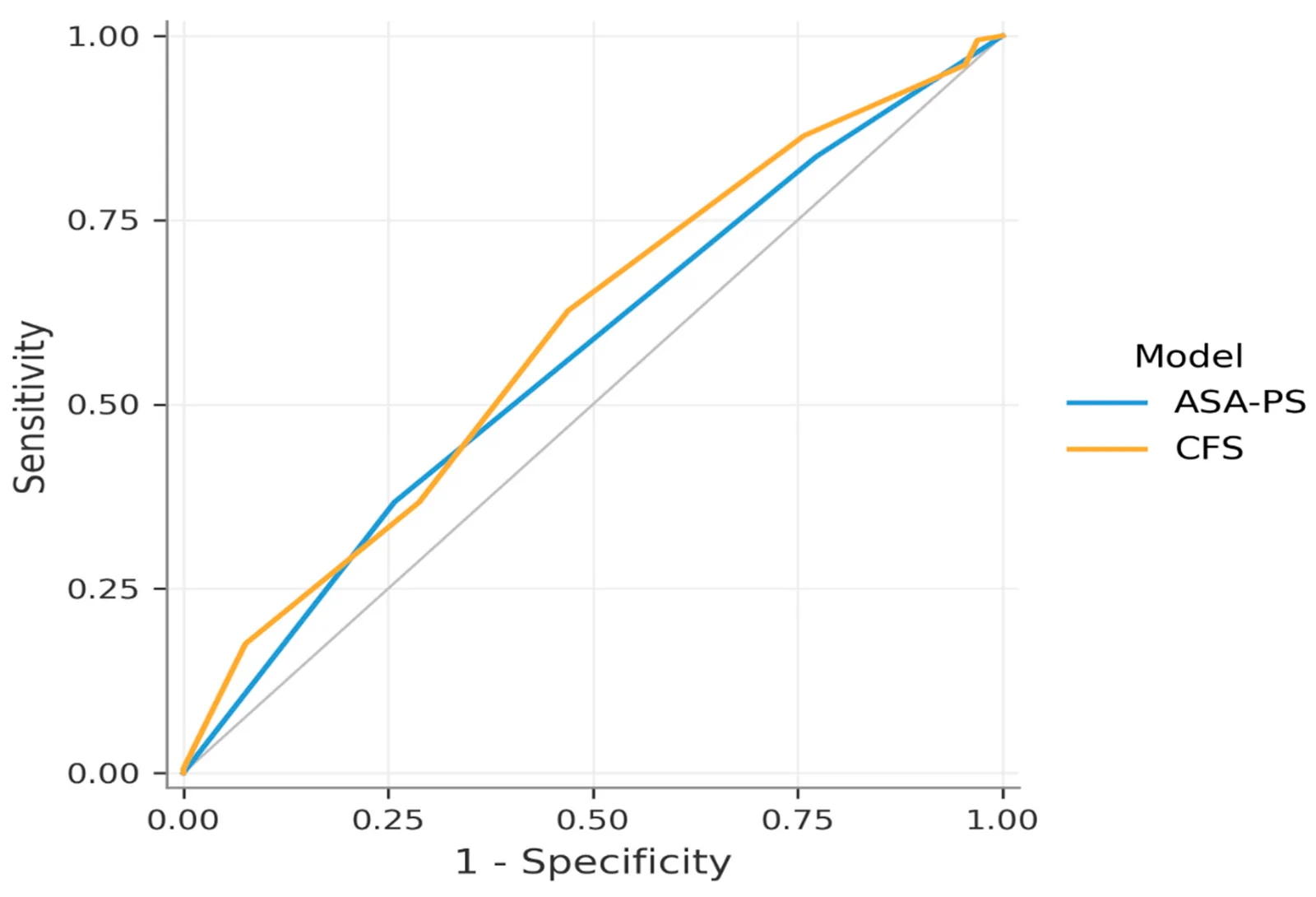
Comparison of Receiver Operating Characteristic (ROC) curves of the Clinical Frailty Scale (CFS) and American Society of Anesthesiologists Physical Status (ASA-PS) score for predicting intensive care unit (ICU) admission. (Note: The blue line represents the ASA-PS score, and the orange line represents the CFS score).

The discriminative performance of both scales in predicting postoperative intensive care unit admission was found to be comparable. While the discriminative power of the CFS was low but statistically significant (AUC: 0.594, 95% CI: 0.516–0.673, *p* = 0.019), the ASA-PS score did not reach statistical significance (AUC: 0.566, 95% CI: 0.492–0.640, *p* = 0.081). Pairwise comparison using the DeLong method showed no significant difference between the ROC curves (AUC difference: −0.028, 95% CI: −0.109 to 0.053; *p* = 0.494). The predictive properties of the scales were presented in Table 3, and the ROC curves were shown in Figure 2.

Subsequently, variables that showed a statistically significant association with ICU admission in univariable analysis age, albumin, hemoglobin, CFS ≥ 5, coronary artery disease, and anesthesia type were included in the multivariable logistic regression model.

On multivariable analysis, age (OR: 1.052; 95% CI: 1.009–1.096; *p* = 0.017) and the presence of coronary artery disease (OR: 3.992; 95% CI: 1.581–10.083; *p* = 0.003) were identified as independent risk factors for postoperative ICU admission. While 83.3% (*n* = 50) of the patients undergoing general anesthesia were admitted to the ICU, 69.4% (*n* = 127) of those undergoing spinal anesthesia required ICU admission. This difference was statistically significant (Fisher’s exact test *p* = 0.044; unadjusted OR = 0.454, 95% CI: 0.215–0.958). After adjustment for age, coronary artery disease, frailty, hemoglobin, and albumin levels, spinal anesthesia was independently associated with lower odds of ICU admission (adjusted OR = 0.363, 95% CI: 0.161–0.823; *p* = 0.015). Variance inflation factor analysis confirmed the absence of multicollinearity among the covariates in the multivariable model (VIF = 1.05) Table 4.

Spearman’s rank correlation analysis revealed a weak-to-moderate positive correlation between ASA-PS and CFS scores (ρ = 0.337; *p* < 0.001), indicating that although these tools share conceptual overlap, they capture partially distinct dimensions of perioperative risk. Post hoc analysis of ASA-PS/CFS concordance across the four subgroups defined above showed no significant between-group difference in ICU admission rate (χ^2^[3] = 6.90, *p* = 0.075; Fisher’s exact *p* = 0.088); detailed subgroup ICU admission rates are presented in Supplementary Table S1.

On univariable logistic regression, CFS as a continuous variable was a significant predictor of in-hospital mortality (OR: 2.79; 95% CI: 1.55–5.04; *p* < 0.001). ROC analysis demonstrated higher discriminatory ability for CFS in predicting in-hospital mortality (AUC: 0.801; 95% CI: 0.693–0.910; *p* < 0.001), with a sensitivity of 84.62% and a specificity of 68.26% at the optimal cut-off value of ≥6. ASA-PS as a continuous variable was not a significant predictor of in-hospital mortality on univariable logistic regression (*p* = 0.317). Given the limited number of mortality events (*n* = 13), a multivariable logistic regression model for in-hospital mortality was not constructed, as the available event count was insufficient to support reliable parameter estimation.

Postoperative delirium occurred in 21 patients (8.6%). Neither ASA-PS nor CFS showed strong discriminative ability for postoperative delirium (AUC 0.611 vs. 0.595, respectively; DeLong *p* = 0.796). Detailed univariable and multivariable regression results are presented in Supplementary Table S2. Acute kidney injury occurred in 42 patients (17.3%), the most frequent postoperative complication in this cohort. On multivariable analysis, GFR was the sole independent predictor of AKI (OR: 0.962; 95% CI: 0.945–0.979; *p* < 0.001); neither CFS (*p* = 0.203) nor ASA-PS retained independent significance after adjustment.

Clinically identified postoperative myocardial injury occurred in 15 patients (6.2%). Neither ASA-PS nor CFS showed discriminative ability for myocardial injury (AUC 0.577 vs. 0.573, respectively; DeLong *p* = 0.973). Given the limited number of events, multivariable regression analysis was not performed for this outcome. Detailed univariable results are presented in Supplementary Table S2.

**Figure 3:**
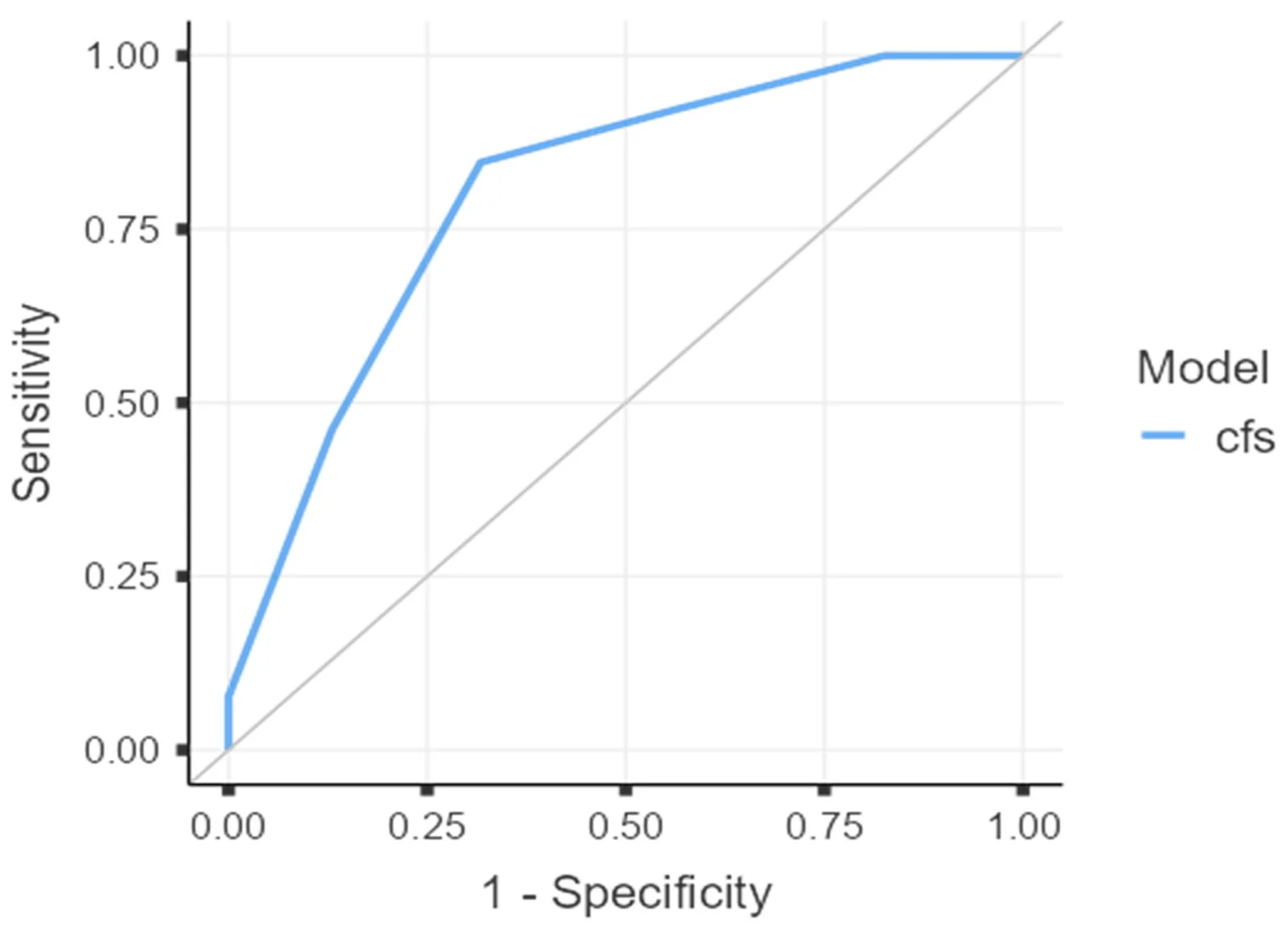
Receiver Operating Characteristic (ROC) curve of the Clinical Frailty Scale (CFS) for predicting in-hospital mortality. The area under the curve (AUC) was 0.801 (95% CI: 0.693–0.910; *p* < 0.001), with an optimal cut-off value of ≥6, yielding a sensitivity of 84.62% and a specificity of 68.26%.

The predictive performance of CFS and ASA-PS for the two clinically most relevant outcomes—ICU admission (Figure 2) and in-hospital mortality (Figure 3)—is summarized in Table 5 and Figure 2 and Figure 3. Detailed regression and association analyses for the remaining secondary outcomes (postoperative delirium, acute kidney injury, and myocardial injury) are presented in Supplementary Table S2.

The median hospital length of stay for the entire cohort was 4.0 days (IQR: 2.0–5.0) and did not differ significantly between ICU-admitted and ward-managed patients (*p* = 0.163); length of stay across other clinical subgroups is presented in Supplementary Table S3.

Neither ASA-PS nor CFS meaningfully improved the discriminative performance of a clinical base model (age, coronary artery disease, anesthesia type) for predicting postoperative ICU admission (AUC 0.709 vs. 0.712 for both; likelihood ratio test *p* = 0.404 and *p* = 0.226, respectively), indicating that neither scoring system contributed independent value beyond established clinical risk factors. Full model details are presented in Supplementary Table S4.

## 4. Discussion

In this cohort, which predominantly consists of high-risk geriatric hip fracture patients, ASA-PS did not retain independent predictive value for any of the outcomes examined in multivariable analysis, despite showing modest univariable discrimination for several secondary outcomes. Regarding postoperative ICU admission, neither scale emerged as an independent predictor; rather, the primary determinants were advanced age, coronary artery disease, and anesthesia type. Furthermore, adding either scoring system to the established base model did not meaningfully improve discriminative performance (AUC: 0.709 vs. 0.712), indicating that frailty assessment provides limited incremental information for acute postoperative ICU need. Given the absence of both independent and incremental predictive value, ASA-PS and CFS alone do not appear to offer meaningful clinical utility for postoperative ICU triage in this high-acuity population. Although the primary objective was to predict ICU admission, an exploratory secondary analysis revealed that CFS demonstrated superior univariable discriminative performance over ASA-PS for in-hospital mortality (AUC: 0.801 vs. 0.623; DeLong *p* = 0.047). Taken together, these results indicate that ICU admission in this group is shaped primarily by perioperative clinical factors, whereas in-hospital mortality risk tracks more closely with baseline physiological reserve—a dimension captured by CFS, but not by ASA-PS.

In the meta-analysis by Dong et al. [9], frail patients demonstrated significantly higher risks of inpatient, 30-day, and 1-year mortality compared with non-frail patients. Similarly, Kennedy et al. [11] reported that increasing CFS scores were associated with higher mortality risk, with CFS and ASA-PS showing comparable discriminative performance for 1-year mortality. In contrast, Ikram et al. [16] observed that the association between CFS and 30-day mortality plateaued beyond a CFS score of 5, displaying only modest discriminative ability. In our cohort, patients with a higher frailty burden (CFS ≥ 6, the mortality-optimal cut-off by ROC analysis) exhibited higher in-hospital mortality, with each one-point increase in CFS score associated with a 2.79-fold increase in univariable mortality odds. Notably, CFS showed higher discriminative performance for in-hospital mortality compared with ASA-PS (AUC: 0.801 vs. 0.623). However, direct comparison with reported AUCs for 30-day (0.63) [16] or 1-year mortality (0.72) [11] is constrained by differences in follow-up timeframes and scoring methodologies; moreover, due to the limited number of events (*n* = 13), a multivariable model could not be constructed; therefore, these mortality findings are based solely on univariable analysis, and independent predictability could not be established, warranting cautious interpretation of these findings. Finally, the limited discriminative power of ASA-PS in our study may be explained by the marked concentration of patients in the ASA-PS III–IV range (81.9%). Similarly, Zaib et al. [17], in a cohort with a comparably skewed ASA-PS distribution, reported that ASA-PS failed to demonstrate meaningful discriminative performance for mortality, while CFS retained strong predictive value.

Frailty prevalence in geriatric hip fracture surgery varies considerably across cohorts, ranging from approximately 48% to 68.5% depending on the assessment tool and population studied [18,19,20]. In our cohort, 58% of patients were classified as frail, which aligns well with the existing literature. Consistent with previous studies evaluating the role of CFS in predicting ICU admission [21], patients admitted to the ICU postoperatively in our study had significantly higher CFS scores. On univariable ROC analysis, CFS demonstrated statistically significant discriminative ability for postoperative ICU admission, whereas ASA-PS did not; however, the DeLong pairwise comparison revealed no significant difference between the two scales. The failure of CFS to retain independent predictive value in multivariable analysis, despite its univariable association with ICU admission, may be explained by several methodological factors. First, the high concentration of ASA-PS III–IV patients (81.9%) may have suppressed the discriminative performance of both scales, as the predictive power of risk models typically diminishes in homogeneous, high-risk cohorts [17,22]. This case-mix homogeneity is also reflected in the exceptionally high ICU admission rate observed in our cohort (72.8%). Furthermore, the weak-to-moderate positive correlation observed between ASA-PS and CFS indicates that these two scales measure related but non-interchangeable constructs; nevertheless, co-including two correlated variables within the same model may have partly attenuated the independent contribution of CFS [11].

The decision to admit a patient to the ICU is not determined solely by preoperative data; intraoperative developments—such as hemodynamic instability, transfusion requirements, or prolonged operative duration—and the individualized judgment of the attending anesthesiologist in response to these events play a decisive role in this process [23,24]. Because such intraoperative events are unavailable to any preoperative scoring system, the loss of independent predictive value for both scales in multivariable analysis aligns logically with the clinical nature of ICU admission decisions. In a high-risk surgical population such as geriatric hip fracture patients, preoperative frailty scoring alone may therefore be insufficient to fully capture the complexity of this decision-making process.

We acknowledge that the exceptionally high ICU admission rate observed in our cohort (72.8%) constitutes a significant limitation regarding the generalizability of our primary outcome. This rate is substantially higher than the 18–29% range commonly reported in the general hip fracture literature [25]; nevertheless, comparably high rates have been documented in similarly high-risk populations, reaching up to 56.2% in an ASA-PS III–IV–predominant cohort in Türkiye [26] and 70% in patients aged over 90 years [13]. These discrepancies suggest that institutional admission policies, rather than patient clinical status alone, may substantially influence ICU utilization rates. Indeed, when compared in terms of age and ASA-PS distribution, Gezer et al., despite finding an ASA-PS III–IV rate of 98.7%—higher than ours (81.9%)—in a group of 450 patients undergoing hip surgery (mean age 82.1 ± 9.0 in those admitted to the ICU), reported an ICU admission rate of only 42.2% [27]. This shows that even when the patient profile is similar or more severe, ICU utilization does not always remain correspondingly high. Similarly, Heuer et al. found that among patients over 90 years of age operated on for hip fracture (mean age 94–95), the ICU admission rate at the same hospital rose from 51% to 70% within a decade [28]. The researchers attributed this increase not to a change in the patients’ risk status, but to the adoption of a more protective triage approach over time and the expansion of intensive care capacity**.** As a single-center tertiary referral institution with a predominantly high-risk case profile (ASA-PS III–IV: 81.9%) and ICU admission practices driven by individual clinician judgment rather than a standardized protocol, our findings regarding ICU admission prediction may not be directly transferable to lower-acuity populations or secondary care hospital settings.

In our study, anesthesia type was independently associated with postoperative ICU admission, with patients undergoing spinal anesthesia having approximately 63.7% lower odds of ICU admission compared to those receiving general anesthesia. When interpreting this finding, a key consideration is that spinal anesthesia may have been preferentially selected for patients with marked hemodynamic frailty or respiratory comorbidities. Since this selection pattern would be expected to bias the association toward higher—rather than lower—ICU admission among spinal anesthesia recipients, the lower observed rate more likely reflects unmeasured intraoperative factors and admission decisions rather than a direct protective effect of spinal anesthesia. The REGAIN trial found no significant difference between spinal and general anesthesia regarding 60-day mortality or ambulation [29]. Furthermore, although general anesthesia has been linked to higher rates of postoperative adverse events, thromboembolic complications, and blood transfusions [30], these mechanisms cannot be disentangled from selection bias in the present observational dataset.

Intraoperative variables such as blood transfusion, hemodynamic instability, and inotrope use were not collected, as the primary aim of this study was to evaluate the predictive performance of preoperatively available frailty and comorbidity indices. However, since anesthesia type itself reflects a perioperative rather than a strictly preoperative decision, this finding should be interpreted with caution rather than as evidence of a causal or purely preoperative effect.

Beyond mortality and ICU admission, the predictive performance of CFS and ASA-PS for postoperative complications remained limited; in multivariable analyses, neither scale was an independent predictor of delirium, AKI, or myocardial injury. Our secondary complication patterns reflect established literature. Dementia emerged as the primary predictor of postoperative delirium [31,32,33], whereas our lower incidence compared to large national registries likely stems from retrospective ICD-10 code retrieval [31]. For acute kidney injury—the most frequent complication—baseline GFR was the sole independent risk factor, reinforcing the importance of preoperative organ reserve. Conversely, the signal linking myocardial injury to younger age contrasts with typical epidemiological patterns [34] and should be viewed as hypothesis-generating given the small event count. Finally, hospital length of stay remained unaffected by ICU admission, but was significantly prolonged by organ-specific complications (Supplementary Table S3).

As an exploratory observation, patients with a low ASA-PS but frail CFS (Group 2) showed an ICU admission rate (75.0%) nearly identical to the concordant high-risk group (Group 3: 75.4%), despite small subgroup sizes (*n* = 12–32) precluding definitive comparison (Supplementary Table S1). This pattern suggests that ASA-PS alone may underrecognize risk in frail patients whose comorbidity-based score is deceptively low, consistent with reports that ASA-PS and CFS provide complementary rather than redundant risk information [9,11,35].

Some methodological limitations of our study should be acknowledged. The single-center, retrospective, and observational design of our study limits the generalizability of our findings. Postoperative ICU admissions reflected the attending anesthesiologist’s intraoperative assessment rather than a fixed institutional criterion, and were additionally shaped by bed availability and local practice patterns; this constrains the evaluation of ASA-PS and CFS as true predictors of ICU admission and should be borne in mind when interpreting our primary findings. Intraoperative variables such as blood transfusion, hemodynamic instability, and inotrope use were not collected, so adjustment could not be made for these factors. As a tertiary referral center with a predominantly high-acuity patient profile (ASA-PS III–IV: 81.9%) and an exceptionally high ICU admission rate (72.8%), the predictive performance values obtained may not fully reflect community hospitals or other healthcare facilities. Regional variations in frailty prevalence, fracture management approaches, and postoperative care pathways are further factors that constrain the external validity of our results. Prospective, multicenter studies incorporating institutions with varying case-mix and ICU admission practices are needed to confirm the generalizability of these findings. The post hoc power analysis for the primary outcome (*n* = 243; 177 ICU vs. 66 non-ICU patients) yielded a statistical power of 93.2% to detect a medium-to-large effect size (δ = 0.50) at a two-tailed alpha level of 0.05, confirming that the sample size was fully adequate. Conversely, due to the lower incidence of secondary endpoints, analyses for these specific outcomes should be interpreted cautiously, as smaller effect sizes may have been undetected. The retrospective ascertainment of CFS from chart documentation may have introduced misclassification bias; however, evaluation by an anesthesiologist blinded to ICU outcomes reduced this risk. The retrospective determination of myocardial injury was based on clinically indicated cardiology consultations rather than routine postoperative troponin monitoring, which likely led to an underestimation of its true incidence. Additionally, mortality was recorded only during the initial hospital stay; post-discharge deaths were not tracked. Postoperative delirium was identified retrospectively through ICD-10 discharge codes and consultation records, which substantially underestimates true incidence; furthermore, the delirium model used only four variables for 21 events (events-per-variable ratio ~5), carrying a risk of overfitting. Finally, due to the small number of events such as myocardial injury (*n* = 15) and mortality (*n* = 13), our findings need to be confirmed in larger cohorts.

## 5. Conclusions

In this study, ASA-PS and CFS demonstrated comparable performance in predicting ICU admission; however, neither scale retained independent predictive value after adjustment for age, coronary artery disease, and type of anesthesia. The high ICU admission rate (72.8%) and the predominance of ASA-PS III–IV patients (81.9%) in our cohort may have contributed to this outcome. Moreover, incorporating either score into the existing clinical risk model did not meaningfully improve discriminative performance (AUC: 0.709 vs. 0.712). Therefore, rather than relying on baseline risk scores for ICU resource planning in this setting, clinical decisions should be guided by comprehensive perioperative evaluation and the intraoperative clinical course. Conversely, CFS exhibited superior discriminative ability compared to ASA-PS for in-hospital mortality. Given the low number of mortality events (*n* = 13), this finding should be interpreted cautiously as exploratory and warrants confirmation in larger, prospective, multicenter studies before a potential role for CFS in mortality risk assessment can be established. From a practical standpoint, incorporating CFS into routine ASA-PS evaluation may contribute to increasing anesthesiologists’ awareness and monitoring of adverse clinical outcomes in high-risk elderly patients, rather than serving as a direct decision criterion for ICU admission.

## Figures and Tables

**Table 1:** Patients’ Demographic, Clinical, and Surgical Characteristics (*n* = 243).

Characteristic	*n* (%)/Value
Sex	
Female	158 (65.0)
Male	85 (35.0)
Frailty (CFS ≥ 5)	
Frail	141 (58.0)
Non-frail	102 (42.0)
ASA-PS	
ASA-PS II	44 (18.1)
ASA-PS III	117 (48.1)
ASA-PS IV	82 (33.7)
Surgical Urgency	
Urgency	184 (75.7)
Emergency	59 (24.3)
Anesthesia Type	
Spinal/Regional	183 (75.3)
General	60 (24.7)
Comorbidities	
Hypertension	133 (54.7)
Diabetes mellitus	81 (33.3)
Chronic kidney disease	62 (25.5)
Coronary artery disease	60 (24.7)
COPD	50 (20.6)
Dementia	44 (18.1)
Congestive heart failure	7 (2.9)
Postoperative Outcomes	
ICU admission	177 (72.8)
Any complication	71 (29.2)
Acute kidney injury (AKI)	42 (17.3)
Delirium	21 (8.6)
Myocardial injury	15 (6.2)
In-hospital mortality	13 (5.3)
Continuous Variables	
Age (years)	81 (75–86)
Operative duration (min)	135 (108–160)
ICU length of stay (days)	1 (0–1)
Albumin (g/L)	35.2 (31.5–38.1)
Creatinine (mg/dL)	0.9 (0.7–1.2)
Hemoglobin (g/dL)	11.1 (9.9–12.6)
ASA-PS score	3 (3–4)
CFS score	6 (5–7)

Categorical variables are presented as *n* (%); continuous variables as median (Q1–Q3). CFS: Clinical Frailty Scale; ASA-PS: American Society of Anesthesiologists Physical Status; COPD: Chronic obstructive pulmonary disease; ICU: Intensive care unit; AKI: Acute kidney injury.

**Table 2:** Comparison of clinical characteristics between patients admitted and not admitted to the ICU.

Variable	ICU Admission		*p*	OR (95% CI)
	No ICU (*n* = 66)	ICU (*n* = 177)		
Demographics				
Age, median (IQR), years	76.5 (70–84)	82.0 (77–87)	**0.001 ^b^**	–
Female sex, *n* (%)	20 (30.3%)	65 (36.7%)	0.351 ^a^	0.75 (0.41–1.38)
Surgical Characteristics				
Emergency surgery, *n* (%)	49 (74.2%)	135 (76.3%)	0.743 ^a^	1.12 (0.58–2.14)
Spinal anesthesia, *n* (%)	56 (84.8%)	127 (71.8%)	**0.044 ^c^**	0.45 (0.21–0.96)
Comorbidities				
Hypertension, *n* (%)	32 (48.5%)	101 (57.1%)	0.232 ^a^	1.41 (0.80–2.49)
Coronary artery disease, *n* (%)	6 (9.1%)	54 (30.5%)	**<0.001 ^a^**	4.39 (1.79–10.8)
Congestive heart failure, *n* (%)	1 (1.5%)	6 (3.4%)	0.678 ^c^	2.28 (0.27–19.3)
Diabetes mellitus, *n* (%)	24 (36.4%)	57 (32.2%)	0.541 ^a^	0.83 (0.46–1.50)
Chronic kidney disease, *n* (%)	16 (24.2%)	46 (26.0%)	0.781 ^a^	1.10 (0.57–2.11)
Alzheimer’s/dementia, *n* (%)	9 (13.6%)	35 (19.8%)	0.349 ^c^	1.56 (0.71–3.45)
CFS score, median (IQR)	5 (4–6)	6 (5–7)	**0.021 ^b^**	–
Frailty (CFS ≥ 5), *n* (%)	31 (47.0%)	110 (62.1%)	**0.041 ^a^**	1.85 (1.05–3.28)
Laboratory Parameters				
Albumin, median (IQR), g/L	37.1 (33.1–40.7)	35.0 (30.0–39.0)	**0.003 ^b^**	–
Hemoglobin, median (IQR), g/dL	11.8 (10.1–13.3)	10.8 (9.6–12.4)	**0.004 ^b^**	–
Creatinine, median (IQR), mg/dL	0.9 (0.72–1.08)	0.92(0.73–1.18)	0.521 ^b^	–
GFR, median (IQR), mL/min/1.73 m^2^	75.0 (57.0–91.5)	64.9 (47.7–87.5)	0.280 ^b^	–
Postoperative Outcomes				
Hospital LOS, median (IQR), days	4.0 (2–5)	4.0 (3–6)	0.163 ^b^	–
In-hospital mortality, *n* (%)	1 (1.5%)	12 (6.8%)	0.105 ^c^	4.73 (0.60–37.1)
Complications, *n* (%)	14 (21.2%)	57 (32.2%)	0.094 ^a^	1.76 (0.90–3.44)

Data are presented as median (interquartile range) for continuous variables and *n* (%) for categorical variables. Bold *p* values indicate statistical significance (*p* < 0.05). For anesthesia type, the reference category is general anesthesia. Abbreviations: ICU, intensive care unit; CFS, Clinical Frailty Scale; OR, odds ratio; CI, confidence interval; GFR, glomerular filtration rate; LOS, length of stay. ^a^ Chi-square test; ^b^ Mann–Whitney U test; ^c^ Fisher’s exact test.

**Table 3:** Predictive performance of ASA-PS and CFS for postoperative ICU admission.

Scale	Optimal Cut-Off	Sensitivity (%)	Specificity (%)	PPV (%)	NPV (%)	AUC	95% CI	*p* Value
	(Youden’s Index)	(95% CI)	(95% CI)					
ASA-PS	≥3.5	36.72 (29.62–44.28)	74.24 (61.99–84.22)	79.27	30.43	0.566	0.492–0.640	0.081
CFS	≥5.5	62.71 (55.14–69.85)	53.03 (40.34–65.44)	78.17	34.65	0.594	0.516–0.673	0.019
DeLong pairwise AUC comparison (ASA-PS vs. CFS): AUC difference = −0.028 (95% CI: −0.109 to 0.053), z = −0.684, *p* = 0.494								

Abbreviations: ASA-PS, American Society of Anesthesiologists Physical Status; CFS, Clinical Frailty Scale; PPV, Positive Predictive Value; NPV, Negative Predictive Value; AUC, Area Under the Curve; CI, Confidence Interval; ICU, Intensive Care Unit.

**Table 4:** Multivariable Logistic Regression Model for Postoperative ICU Admission.

Variable	B	SE	Wald	*p*-Value	OR	95% CI
Age (years)	0.051	0.021	5.659	**0.017**	1.052	1.009–1.096
Frailty (CFS ≥ 5 vs. <5)						
Yes vs. No	0.196	0.332	0.348	0.555	1.216	0.634–2.332
Coronary artery disease						
Yes vs. No	1.384	0.473	8.580	**0.003**	3.992	1.581–10.083
Hemoglobin (g/dL)	−0.140	0.088	2.500	0.114	0.870	0.731–1.034
Albumin (g/L)	−0.040	0.034	1.402	0.236	0.961	0.899–1.027
Anesthesia type						
Spinal vs. General	−1.012	0.417	5.890	**0.015**	0.363	0.161–0.823

Binary logistic regression was performed using age as a continuous variable. OR represents the odds ratio for postoperative ICU admission. Reference categories: frailty absent (CFS < 5), no coronary artery disease, general anesthesia. Wald statistic = Z^2^. Bold *p*-values indicate statistical significance (*p* < 0.05). B: regression coefficient; SE: standard error; OR: odds ratio; CI: confidence interval; CFS: Clinical Frailty Scale; ICU: intensive care unit.

**Table 5:** ROC analysis and pairwise comparison of ASA-PS and CFS for ICU admission and in-hospital mortality.

Outcome	CFS			ASA-PS			AUC Difference	DeLong *p*
	AUC	95% CI	*p*	AUC	95% CI	*p*		
ICU admission	0.594	0.516–0.673	0.019	0.566	0.492–0.640	0.081	−0.028	0.494
Mortality	0.801	0.693–0.910	<0.001	0.623	0.510–0.736	0.046	+0.178	0.047 *

* *p* < 0.05 statistically significant; ICU: intensive care unit; CI: confidence interval; DeLong: pairwise AUC comparison using DeLong’s method.

## Data Availability

The data presented in this study are available on reasonable request from the corresponding author. The data are not publicly available due to privacy and ethical restrictions.

## Supplementary Materials

The following supporting information can be downloaded at: https://www.mdpi.com/article/10.3390/jcm15166263/s1, Table S1. Postoperative ICU admission rates according to ASA-PS/CFS concordance–discordance subgroups. Table S2. Univariable and multivariable logistic regression analyses for postoperative complications. Table S3. Hospital length of stay according to postoperative complication status. Table S4. Hierarchical logistic regression models for postoperative ICU admission: incremental value of ASA-PS and CFS beyond the clinical base model.

## Abbreviations

AKI	Acute Kidney Injury
ASA-PS	American Society of Anesthesiologists Physical Status
AUC	Area Under the Curve
CFS	Clinical Frailty Scale
GFR	Glomerular Filtration Rate
ICD-10	International Classification of Diseases
ICU	Intensive Care Unit
