# Supplementary material for: Comparative Analysis of the ASA-PS Score and Clinical Frailty Scale in Predicting Postoperative Intensive Care Unit Requirement in Geriatric Hip Fracture Surgery: A Retrospective Evaluation
Source: J Clin Med. 2026 Aug 13;15(16):6263. doi: 10.3390/jcm15166263 (PMC13513097; doi:10.3390/jcm15166263)
Supplement: Supplementary file 1 [file jcm-15-06263-s001.zip › jcm-4462229-supplementary.pdf]

Table S1. *Postoperative ICU admission rates according to ASA-PS/CFS concordance–discordance subgroups.*

	Definition	n (%)	ICU admission, n (%)
1	ASA-PS 2, non-frail CFS	12 (4.9)	5 (41.7)
2	ASA-PS 2, frail CFS	32 (13.2)	24 (75.0)
3	ASA-PS 3–4, frail CFS	171 (70.4)	129 (75.4)
4	ASA-PS 3–4, non-frail CFS	28 (11.5)	19 (67.9)

ASA-PS: American Society of Anesthesiologists Physical Status; CFS: Clinical Frailty Scale; ICU: intensive care unit. Frail was defined as CFS ≥5. Between-group difference in ICU admission rate: $\chi^2(3) = 6.90$, $p = 0.075$; Fisher's exact $p = 0.088$.

Table S2. *Univariable and multivariable logistic regression analyses for postoperative complications*.

Variable	Univariable Analysis			Multivariable Analysis		
	OR	95% CI	p	OR	95% CI	p
**Postoperative Delirium (n=21, 8.6%)**						
Age (years)	1.08	1.02–1.15	0.014	1.043	0.977–1.113	0.203
Hemoglobin (g/dL)	0.776	0.616–0.978	0.032	0.841	0.647–1.094	0.195
Albumin (g/L)	0.901	0.830–0.978	0.013	0.937	0.850–1.033	0.193
Dementia	5.027	1.981–12.753	<0.001	3.570	1.314–9.700	0.013*
**Acute Kidney Injury (n=42, 17.3%)**						
CFS (per unit)	1.520	1.170–1.974	0.002	1.209	0.903–1.620	0.203
Emergency surgery	0.388	0.192–0.785	0.008	0.490	0.224–1.071	0.074
Hemoglobin (g/dL)	0.843	0.711–0.999	0.048	0.944	0.774–1.153	0.575
GFR (mL/min/1.73m²)	0.957	0.941–0.973	<0.001	0.962	0.945–0.979	<0.001*
**Clinically Identified Postoperative Myocardial Injury (n=15, 6.2%)**						
Age (years)	0.926	0.862–0.994	0.034			
Female sex	0.333	0.114–0.971	0.044			
Emergency surgery	0.338	0.117–0.975	0.045			

OR: odds ratio; CI: confidence interval; CFS: Clinical Frailty Scale; GFR: glomerular filtration rate; Only variables significant in univariable analysis (p<0.05) were entered into multivariable models. Emergency surgery was coded as 0 (emergency) vs. 1 (urgency); accordingly, OR<1 indicates higher risk associated with emergency surgery

Table S3. *Hospital length of stay according to postoperative complication status.*

Complication	Absent, median (IQR), days	Present, median (IQR), days	p
Acute kidney injury	4.0 (2.0–6.0)	5.5 (3.0–8.0)	0.010
Postoperative delirium	4.0 (2.0–7.0)	5.0 (3.0–8.0)	0.180
Myocardial injury	4.0 (2.0–7.0)	4.0 (2.0–9.0)	0.650
Any complication (overall)	4.0 (2.0–6.0)	5.0 (3.0–9.0)	0.002*

*IQR: interquartile range. p-values from Mann-Whitney U test, given the non-normal distribution of length of stay (Shapiro-Wilk $p < 0.001$). Hospital length of stay did not differ significantly between ICU-admitted and ward-managed patients (median 4.0 [2.0–7.0] vs. 4.0 [2.0–5.0] days; $p = 0.163$; see Table 2). * $p < 0.05$, statistically significant.*

Table S4. *Hierarchical logistic regression models for postoperative ICU admission: incremental value of ASA-PS and CFS beyond the clinical base model.*

Model	Variables added	AUC	LR χ^2 (df)	P
Base model	Age, coronary artery disease, anesthesia type	0.709	—	—
Base + ASA-PS	+ ASA-PS	0.712	0.697 (1)	0.404
Base + CFS	+ CFS	0.712	1.47 (1)	0.226
